# Multi-layered mutation in hedgehog-related genes in Gorlin syndrome may affect the phenotype

**DOI:** 10.1371/journal.pone.0184702

**Published:** 2017-09-15

**Authors:** Shoko Onodera, Akiko Saito, Daigo Hasegawa, Nana Morita, Katsuhito Watanabe, Takeshi Nomura, Takahiko Shibahara, Shinsuke Ohba, Akira Yamaguchi, Toshifumi Azuma

**Affiliations:** 1 Department of Biochemistry, Tokyo Dental College, Tokyo, Japan; 2 Division of Clinical Biotechnology, The University of Tokyo Graduate School of Medicine, Bunkyo-ku, Tokyo, Japan; Department of Bioengineering, The University of Tokyo Graduate School of Engineering, Bunkyo-ku, Tokyo, Japan; 3 Department of Oral and Maxillofacial Surgery, Tokyo Dental College, Tokyo, Japan; 4 Department of Oral Medicine, Oral and Maxillofacial Surgery, Tokyo Dental College, Chiba, Japan; 5 Oral health science center, Tokyo Dental College, Tokyo, Japan; CNR, ITALY

## Abstract

Gorlin syndrome is a genetic disorder of autosomal dominant inheritance that predisposes the affected individual to a variety of disorders that are attributed largely to heterozygous germline patched1 (*PTCH1*) mutations. PTCH1 is a hedgehog (Hh) receptor as well as a repressor, mutation of which leads to constitutive activation of Hh pathway. Hh pathway encompasses a wide variety of cellular signaling cascades, which involve several molecules; however, no associated genotype–phenotype correlations have been reported. Recently, mutations in Suppressor of fused homolog (*SUFU*) or *PTCH2* were reported in patients with Gorlin syndrome. These facts suggest that multi-layered mutations in Hh pathway may contribute to the development of Gorlin syndrome. We demonstrated multiple mutations of Hh-related genes in addition to *PTCH1*, which possibly act in an additive or multiplicative manner and lead to Gorlin syndrome. High-throughput sequencing was performed to analyze exome sequences in four unrelated Gorlin syndrome patient genomes. Mutations in *PTCH1* gene were detected in all four patients. Specific nucleotide variations or frameshift variations of *PTCH1* were identified along with the inferred amino acid changes in all patients. We further filtered 84 different genes which are closely related to Hh signaling. Fifty three of these had enough coverage of over ×30. The sequencing results were filtered and compared to reduce the number of sequence variants identified in each of the affected individuals. We discovered three genes, *PTCH2*, *BOC*, and *WNT9b*, with mutations with a predicted functional impact assessed by MutationTaster2 or PolyPhen-2 (Polymorphism Phenotyping v2) analysis. It is noticeable that PTCH2 and BOC are Hh receptor molecules. No significant mutations were observed in *SUFU*. Multi-layered mutations in Hh pathway may change the activation level of the Hh signals, which may explain the wide phenotypic variability of Gorlin syndrome.

## Introduction

Gorlin syndrome, also referred to as the nevoid basal cell carcinoma syndrome (NBCCS; OMIM 109400), is characterized by several craniofacial abnormalities such as, macrocephaly, frontal bossing and coarse facial features. Recurrent pathological features, such as the development of basal cell carcinomas (BCCs) and odontogenic keratocysts, is a prominent characteristic of these patients[1,2]. Most individuals have skeletal anomalies (e.g., bifid ribs and wedge-shaped vertebrae). Ectopic calcification, particularly in the cerebral falx, is present in more than 90% of the affected individuals. Approximately 5% of all children with Gorlin syndrome develop medulloblastoma (primitive neuroectodermal tumor [PNET]), usually of the desmoplastic subtype. The condition has an autosomal dominant inheritance. Mutations affecting the transmembrane protein patched homolog 1 (*PTCH1*) are most frequently observed[3–7]. Additionally, mutation in a protein suppressor of fused (SUFU), encoded by *SUFU* gene[8,9], part of a corepressor complex, and *PTCH2* are reported[10,11].

Heterozygous germline *PTCH1* mutations have been detected in patients with Gorlin syndrome and the loss of function *PTCH1* mutations are predicted to reduce suppression of Smoothened (SMO). In vertebrates, intracellular signaling activity is mediated through the zinc finger transcription factors GLIs. Suppression of SMO leading to GLI repressor formation was shown to block trans-localization of GLI into nucleus [12,13]. Mutation in *PTCH1* gene causes functional loss of PTCH1 protein and gives rise to constitutive activation of hedgehog (Hh) signaling[14,15]. Hh signaling pathway governs complex developmental processes, including proliferation and patterning within diverse tissues including osteogenesis. These activities rely on a tightly regulated transduction system that converts graded Hh input signals into specific levels of pathway activity.

Recently, several other Hh receptors have been reported. Hedgehog-interacting protein 1 (HHIP1) is a Hh receptor whose function is similar to that of PTCH1 and PTCH2, i.e., to suppress Hh signaling[16]. Three other Hh co-receptors have been reported. CAM-related/down-regulation by oncogenes (CDON), brother of CDON (BOC), and growth arrest-specific 1 (GAS1) are reported as positive transducers of Hh signaling during embryonic neuronal development [17,18]. However, the potential biological role of these co-receptors in skeletal development has not been reported.

The development of high-throughput methods, i.e., next generation sequencing (NGS), has enabled genome-wide characterization of mutations. Several mutations in *PTCH1* have been reported that mention it as the causative gene of Gorlin syndrome by conventional Sanger sequencing. However, Sanger sequencing of *PTCH1* sometimes fails to determine mutations, probably because of technical problems[19–21]. Although the analysis of large genes such as *PTCH1* is technically challenging, few reports have described the genome-wide characterization of mutation analysis of Gorlin syndrome. Accurate deleteriousness prediction for variants is crucial to screen out pathogenic mutations from background polymorphisms in NGS studies. Computational algorithms are key inputs for the characterization of variants owing to their ability to be employed on a scale that is consistent with the large number of variants being identified from the systematic screening of representative human populations [22].

In this context several sequence- and/or structure-based methods have been used to predict the potential impact of amino acid substitutions on protein structure and activity. PolyPhen-2[23], MutationTaster2 [24,25], and others are able to predict 90% or more of the damaging mutations. For a given amino acid substitution in a protein, PolyPhen-2 extracts various sequence- and structure-based features of the substitution site and feeds them to a probabilistic classifier.[23] These prediction programs allow efficient filtering out of alterations that have a high disease-causing potential from the NGS data. MutationTaster2 integrates information from different biomedical databases and utilizes established analytic tools. Components of the analyses include evolutionary conservation and footprints. These prediction methods can help us narrow down candidate mutations and help identify the causative genes. Since these programs have both advantages and disadvantages, we tried PolyPhen-2 and MutationTaster2 to narrow down the candidate mutations. Here we report NGS performed to detect mutations which might cause functional alterations in Hh signaling. We found mutations in PTCH1 in all four patients, with additional mutations in several molecules involved in Hh signaling. Two of these pertained to Hh receptor PTCH2 and BOC. MutationTaster2 analysis indicates that these mutations may cause functional alteration.

## Materials and methods

### Ethics statement

Written informed consent was obtained from all participants for this molecular genetics study. The study was approved by the Ethics Committee for clinical research at the Tokyo Dental College (Tokyo Japan) (no.527 and no.575) and complies with the tenets of the declaration of Helsinki.

### Cell culture

Gorlin syndrome fibroblasts were obtained from oral tissues resected as part of treatment for diseases. Fibroblasts were collected from unaffected oral mucosa at the time of surgical operation. Fibroblasts were cultured in Dulbecco’s modified Eagle’s medium (DMEM) (Invitrogen, Carlsbad, CA, USA) supplemented with 10% fetal bovine serum (FBS) (Invitrogen, Carlsbad, CA, USA) and 1% Penicillin/Streptomycin (Invitrogen, Carlsbad, CA, USA) and maintained at 37°C in 5% CO_2_.

### Sequencing and data analyses

Genomic DNA was extracted using an Easy-DNA™ gDNA Purification Kit (Life technology). Genomic DNA was fragmented by Covaris. The size of the library fragments was mainly distributed between 200 and 250 bp. Enrichment of coding exons was performed using Sure Select XT Human All Exon v5 kit (Agilent Technologies, California, USA) for generation of exome libraries. The enriched exome was sequenced on Illumina Hiseq 2500 (Illumina Inc., San Diego, CA, USA) with 101-bp paired-end reads, which were aligned to the human reference genome (UCSC, hg19/GRCh37) with Burrows-Wheeler Alignment software (version 0.7.10, http://bio-bwa.sourceforge.net/) for the subsequent variant analysis. Samtools (version 1.1, http://www.htslib.org/man/samtools/) and Picard tools (version 1.115, http://broadinstitute.g.,ithub.io/picard/) were used to build indices and eliminate duplicates. Local realignment around indels (RealignerTargetCreator, IndelRealigner) and base score recalibration (BaseRecalibrator) were applied by GATK (The Genome Analysis ToolKit, Lite version 2.3.0) to ensure accuracy in the identification of indels and single nucleotide variants (SNVs). For each sample, a processed alignment file was saved in bam form. Previously identified common variants (frequency > 1%) and synonymous substitutions were filtered out using public databases including dbSNP 142, HapMap samples, and the 1000 Genome Project (http://www.1000genomes.org). The sequencing data were available in the DDBJ databank of Japan (Accession number: JGAS00000000099).

### Validation analysis of mutations

**Fig 1:**
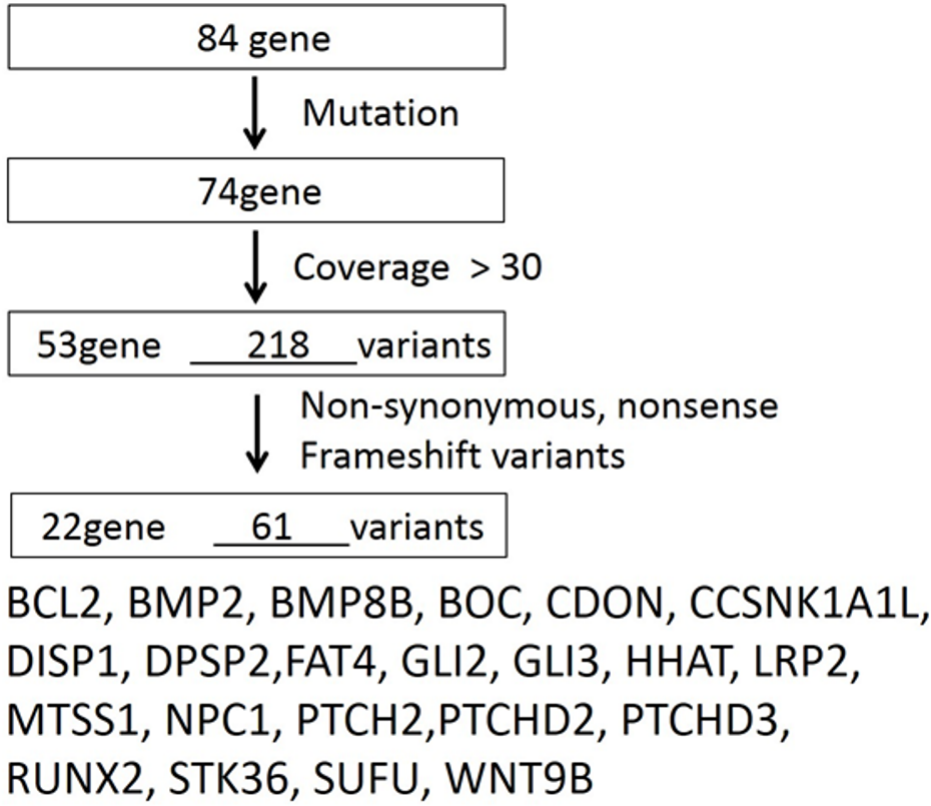
Flow chart indicating the validation process for variants. After the validation steps, 61 variations were selected.

Data were analyzed using SAM tools and SnpEff (http://snpeff.sourceforge.net/index.html). The mutations were confirmed using the Integrative Genomics Viewer (IGV) browser. The flow chart used to select candidate genes is shown in Fig 1. To identify pathogenic DNA sequence alterations, we used MutationTaster2 (http://www.mutationtaster.org/index.html) [25,26] and PolyPhen-2 v2.2.2r398 (http://genetics.bwh.harvard.edu/pph2/index.shtml) [27,28]. MutationTaster2 grades the mutations as “disease causing automatic” (deleterious); “disease causing” (probably deleterious); “polymorphism” (probably harmless); and “polymorphism automatic” (harmless).

The PolyPhen-2 grades the mutations as probably damaging, possibly damaging, or benign, along with a numerical score ranging from 0.0 (benign) to 1.0 (damaging). Though the possibly damaging score is generally interpreted as an indication of a mild effect or low penetrance, it is intended as a measure of prediction confidence rather than the effect size. Identified variants were also compared with known alterations reported in mutation databases such as the Human Gene Mutation Database (HGMD).

## Results

### Mutations in *PTCH1*

**Fig 2:**
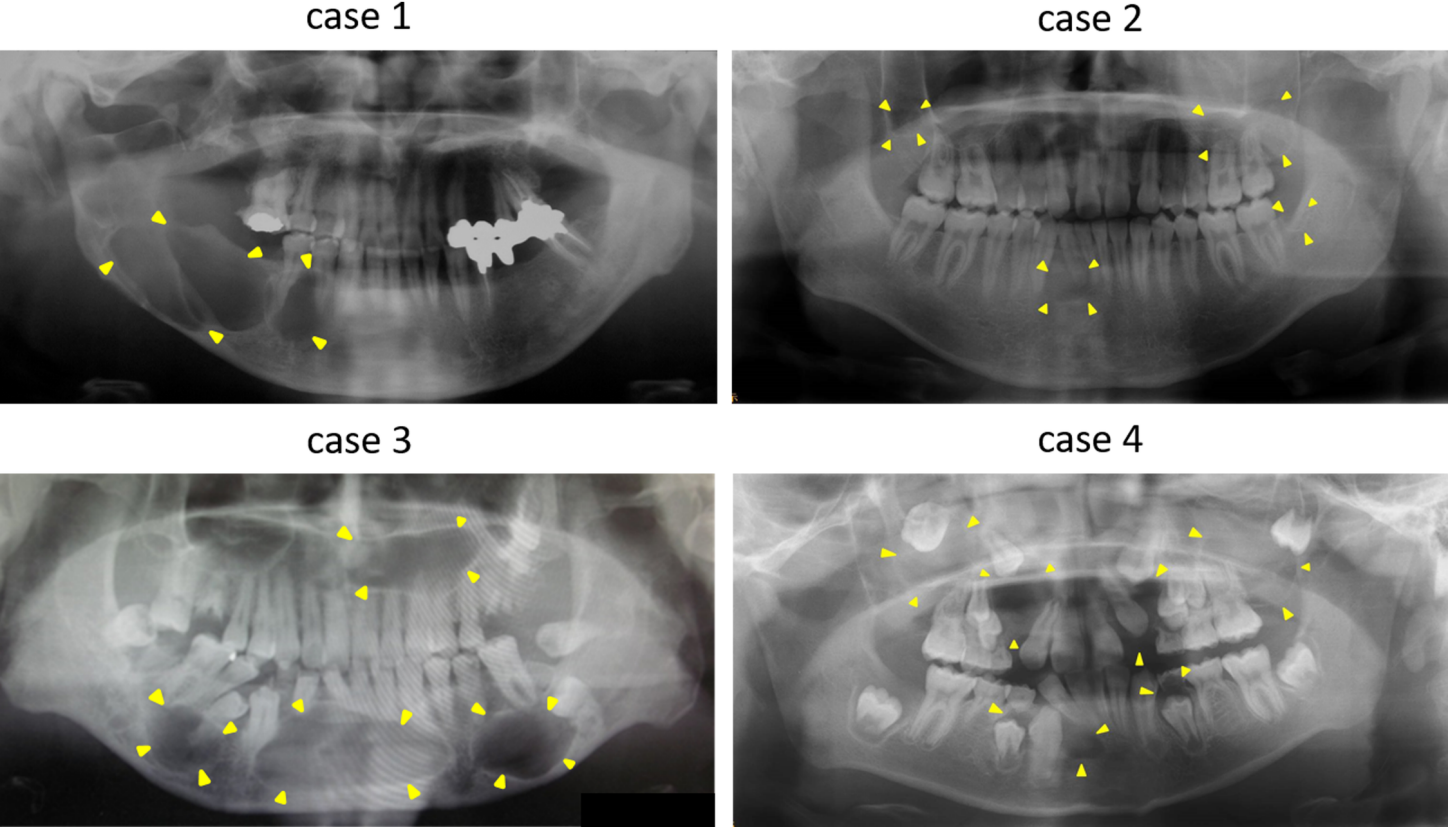
Panoramic radiograph demonstrates the presence of odontgenic keratocysts. Radiologic images showed odontogenic keratocystes in each individual. Yellow arrows showed radiolucent lesions.

Clinical features of the four Gorlin syndrome patients enrolled in this study are summarized in Table 1. We used the criteria described by Kimonis et al., which comprises of six major and six minor criteria[29], for the diagnosis of Gorlin syndrome. All four patients met at least two major criteria. All individuals had odontogenic keratocysts (Fig 2) and had a familial history of the syndrome. Case 1 had BCCs, skin pit, spinal deformity, and calcification of the falx. Case 2 had BCCs and skin pit. Case 3 showed skin pit, rib anomaly, and calcification of the falx. Case 4 only had KCOT and family history.

**Table 1:** Six major criteria by Kimonis and the PTCH1 mutation in the four study subjects with Gorlin syndrome.

case	birth date	sex	mutation	phenotype					
				KCOT	BCC	skin pit	skeletal anomaly	calcification of the falx cerebri	family with Gorlin S
1	1953	M	frameshift: p.Leu446fs	+	+	+	+ (spinal deformity)	-	+
2	1987	M	splice_donor_variant: c.652G>A	+	+	-	-	-	+
3	1983	M	frameshift: p.Asp460fs	+	-	+	+ (rib anomaly)	+	+
4	1995	M	missense: p.Leu505Arg	+	-	-	-	-	+

The six major criteria include 1) more than 2 BCCs or one under the age of 20 years; 2) odontogenic keratocysts of the jaw proven by histology; 3) three or more palmar or plantar pits; 4) bilamellar calcification of the falx cerebri; 5) bifid, fused, or markedly splayed ribs; and 6) first degree relative with NBCC syndrome.

To identify the alterations in *PTCH1*, mutation analysis was performed using MutationTaster2. We observed an average of 95.33% of bases at >30X coverage from exome sequence. Specific SNVs causing inferred amino acid changes were detected in two patients and frameshift mutations were observed in two patients.

Case 1: we identified p.Leu446 frameshift mutation located in exon 9. A previous report showed similar alteration showing only one amino-acid change [30] located in the 2^nd^ transmembrane domain (p.438–457).

Case 2: This c.652G>A alteration occurred at a 5′splice site of exon–intron boundaries, which probably led to the exon skipping and intron retention in the mRNA transcript, resulting in the abnormal protein formation.

Case 3: We identified a p.Asp460 frameshift mutation located in exon 10. p.Asp460 mutation was close to the 2^nd^ transmembrane domain (p.438–457).

Case 4: The p.Leu505Arg changes were predicted to be functionally pathogenic by MutationTaster2, and have not been reported in either HGVD. p.Leu505Arg mutation was located near the 4^th^ transmembrane domain (p.502–522).

We found mutations in the *PTCH1* gene at the transmembrane domain or a close-by site in all patients and these mutations have never been reported.

### Additional mutations were discovered in Hh-related genes

Next we focused on the 84 Hh-related genes listed in Table 2. To discriminate mutations from polymorphisms, we narrowed down our search as illustrated in the flowchart (Fig 2). Specific SNVs causing inferred amino acid changes were detected in 22 genes (Table 3). Each individual had different types of mutations in eight genes, *BMP2*, *CDON*, *CSNK1A1L*, *DISP2*, *FAT4*, *LRP2*, *NPC1*, and *PTCHD3*, but all patients had at least one of these additional mutations.

**Table 2:** Gene list for 84 genes.

gene symbol	function								
	1	2	3	4	5	6	7	8	9
BOC	**+**								
BCL2					**+**				
BMP2					**+**	**+**			
BMP4					**+**	**+**			
BMP5					**+**	**+**			
BMP6					**+**	**+**			
BMP7					**+**	**+**			
BMP8B					**+**	**+**			
BTRC (bTrCP)			**+**				**+**		
CDON	**+**								
CSNK1A1			**+**						
CSNK1E			**+**						
CTNNB1							**+**		
DHH	**+**								
DISP1				**+**					
DISP2				**+**					
ERBB4									**+**
FAT4								**+**	
FBXW11			**+**				**+**		
FGF9				**+**			**+**		
FGFR3									**+**
FKBP8				**+**					
FOXE1									**+**
FRMD6								**+**	
GAS1	**+**								
GLI1			**+**						
GLI2			**+**						
GLI3			**+**						
GREM1						**+**			
GSK3B			**+**				**+**		
HHAT				**+**					
HHIP	**+**								
IFT52				**+**					
IHH	**+**								
KCTD11				**+**					
LATS1							**+**	**+**	
LATS2							**+**	**+**	
LRP2		**+**							
MAPK1 (ERK2)									**+**
MOB1B								**+**	
MTSS1					**+**				
NF2								**+**	
NPC1				**+**					
NUMB									**+**
OTX2				**+**					
PRKACA			**+**						
PRKACB			**+**						
PTCH1		**+**			**+**				
PTCH2		**+**			**+**				
PTCHD1		**+**							
PTCHD2		**+**							
PTCHD3		**+**							
RAB23		**+**							
RUNX2									**+**
SFRP1						**+**			
SHH	**+**								
SMO		**+**							
STK3								**+**	
STK36			**+**						
SUFU			**+**						
TP53 (p53)								**+**	
VEGFA					**+**				
WIF1							**+**		
WNT1					**+**		**+**		
WNT10A					**+**		**+**		
WNT10B					**+**		**+**		
WNT11					**+**		**+**		
WNT16					**+**		**+**		
WNT2					**+**		**+**		
WNT2B					**+**		**+**		
WNT3					**+**		**+**		
WNT3A					**+**		**+**		
WNT4					**+**		**+**		
WNT5A					**+**		**+**		
WNT5B					**+**		**+**		
WNT6					**+**		**+**		
WNT7A					**+**		**+**		
WNT7B					**+**		**+**		
WNT8A					**+**		**+**		
WNT8B					**+**		**+**		
WNT9A					**+**		**+**		
WNT9B					**+**		**+**		
ZIC1			**+**						
ZIC2 (HPE5)			**+**						

It was classified by the action of the Hedgehog-related gene. Numbers 1–5 were related to hedgehog signaling. 1: hedgehog ligands and regulators; 2: hedgehog receptors and cofactor; 3: transcription factors and regulators; 4: other hedgehog related signaling genes; 5: hedgehog signaling target genes. Numbers 6–9 were related to pathways cross talking with hedgehog signaling; 6: TGF beta signaling; 7: WNT signaling; 8: Hippo signaling; and 9: other hedgehog signaling genes.

**Table 3:** Multi-layered mutations were observed in the four study subjects with Gorlin syndrome.

gene	case.1	case.2	case.3	case.4
BCL2				**+**
BMP2	**+**	**+**	**+**	**+**
BMP8B	**+**	**+**		**+**
BOC		**+**	**+**	**+**
CDON	**+**	**+**	**+**	**+**
CSNK1A1L	**+**	**+**	**+**	**+**
DISP1	**+**	**+**		**+**
DISP2	**+**	**+**	**+**	**+**
FAT4	**+**	**+**	**+**	**+**
GLI2	**+**	**+**	**+**	
GLI3		**+**		
HHAT	**+**		**+**	**+**
LRP2	**+**	**+**	**+**	**+**
NPC1	**+**	**+**	**+**	**+**
MTSS1				**+**
PTCH2	**+**		**+**	
PTCHD2	**+**		**+**	**+**
PTCHD3	**+**	**+**	**+**	**+**
SUFU	**+**	**+**	**+**	**+**
RUNX2			**+**	
STK36			**+**	**+**
WNT9B		**+**		
total number (84)	15	15	16	17

After the validation steps shown in Fig 2, 22 gene alterations were selected from 4 individuals.

First, we investigated whether mutations were located in either the *PTCH2* or *SUFU* genes, since both were reported as rare causative genes of Gorlin syndrome. We identified p.Arg175Leu and p.Arg74His in case 1 and p.Arg673Leu in case 3 in the *PTCH2* gene (Table 4). P.Arg175Leu and p.Arg74His were categorized as “disease causing” by MutationTaster2 and as “probably damaging” and “possibly damaging” by Polyphen-2. We detected c.1299T>C in all individuals in *SUFU*. Although c.1299T>C in *SUFU* was categorized as “polymorphism” by MutationTaster2, we qualified this alteration as non-causative.

**Table 4:** Disease-causing mutated gene.

case	SNP	Gene	nucleoside acid change	type	AA change	Exon number	MAF	MutationTaster2	polyphen2
1	rs76491994	'FAT4	5698A>C	MISSENSE	Ile1900Leu	9	C = 0.0096/48	disease causing	benign (0.010)
		'PTCH2	524G>T	MISSENSE	Arg175Leu	4		disease causing	probably damaging (0.990)
		'PTCH2	221G>A	MISSENSE	Arg74His	2		disease causing	possibly damaging (0.993)
2	rs149931425	'WNT9B	773G>A	MISSENSE	Arg258His	4	A = 0.0010/5	disease causing	possibly damaging (0.579)
3		'PTCH2	2018G>T	MISSENSE	Arg673Leu	14		disease causing	probably damaging (0.990)
	rs535015975	'RUNX2	85T>C		Phe29Leu	1	C = 0.0004/2	disease causing	
	rs3814400	BOC	925G>A	MISSENSE	Gly309Arg	7	A = 0.0020/10	disease causing	probably damaging (0.998)
4	rs3814404	BOC	2737C>T	MISSENSE	Pro913Ser	17	T = 0.0076/38	disease causing	benign (0.007)

From 22 genes, disease-causing mutated genes were selected by prediction problem, MutationTaster2, and PolyPhen2.

Second, to identify the impact of an amino acid substitution on the structure or function of proteins expressed in patients with inherited diseases, we analyzed 22 genes with 61 variants using MutationTaster2 and PolyPhen-2.

Case 1: We identified mutations in 15 genes (Table 3). p.Ile1900Leu in *FAT4* (FAT atypical cadherin 4) was predicted as functionally pathogenic by MutationTaster2 (Table 4).

Case 2: We identified mutations in 15 genes (Table 3). The mutation located in *WNT9B* was predicted to be functionally pathogenic. The p Arg258His in *WNT9B* was located on chromosome 17q21. WNT9b is expressed in the ectoderm of the early facial prominence in mice [31,32]. The p Arg258His mutation was predicted to be “possibly damaging” by polyphen-2 (Table 4).

Case 3: We identified mutations in 16 genes (Table 3). Among these, mutations in RUNX2 and *BOC* were identified as disease causing genes. The p.Phe29Leu in intron of *RUNX2* gene was predicted to be “disease causing” by MutationTaster2. p.Gly309Arg was detected in *BOC*, one of the Hh receptors, which regulates Shh-dependent cell fate specification and axon guidance[17]. The p.Gly309Arg mutation was predicted to be “probably damaging (0.998)” by PolyPhen-2.

Case 4: We identified mutations in 17 genes (Table 3). p.Pro913Ser was detected in *BOC*. This mutation was predicted as “disease causing” by MutationTaster2. (Table 4)

## Discussion

We present here two major findings. First, the four individual patients who qualified the diagnostic criteria for Gorlin syndrome had novel *PTCH1* gene mutations. These mutations were located near transmembrane lesions. Second, exome analysis of all four patients revealed additional mutations in Hh receptor *PTCH2* or *BOC*.

Whole-exome sequencing (WES) has become the standard method for identification of causative genes of rare monogenic diseases [33,34]. An efficient and popular strategy is “intersection filtering,” in which the whole-exomes of several unrelated affected individuals are sequenced, and their sequence variants are filtered and compared[35]. This approach is useful for both rare inherited and sporadic diseases. We performed an exome analysis to detect mutations in the predicted gene *PTCH1*, in patients with Gorlin syndrome. Although numerous germline mutations of *PTCH1* have been reported in these patients, neither clustering of mutations nor genotype–phenotype correlations have been identified. However, the onset of related malignancies in patients with Golrin syndrome is based on the classical 2 hit suppressor gene model: baseline heterozygosity preceding the germline PTCH1 mutation is the first hit. The second hit is UV or ionizing radiation.

Congenital malformations may be attributable to alterations in the concentration of the *PTCH1* gene product in the dosage-sensitive Hh signaling pathway. Therefore, it is highly likely that genetic variations contributed to PTCH1 phenotype variations. Other important facts of genetic analysis of Gorlin syndrome was that many cases that researchers cannot find mutations in the *PTCH1* gene probably due to technical difficulties or existence of mutations outside of the regions analyzed or in genes other than *PTCH1* [21]. These facts suggest the need to consider possible mutations in genes other than *PTCH1*.

In fact, *SUFU* [8] and *PTCH2* [10,11] mutations have been reported as another possible causative genes of Gorlin syndrome. However, few studies have investigated *PTCH1* and other possible mutations simultaneously.

Because increasingly sophisticated methods particularly emphasize the fact that primary mutation discovery in humans will remain crucial to scientific progress, analysis of the WES and whole-genome sequencing (WGS) data allows for determination of the phenotypic consequences of genetic variation. Therefore, it is reasonably assumed that the mutation analysis of genes other than *PTCH1* using WES may define potential diagnostic, preventive, and therapeutic opportunities for Gorlin syndrome and illuminate disease mechanisms.

In this study, we first used NGS to screen *PTCH1* genes in four individuals with Gorlin syndrome. We detected four novel mutations, of which p.Leu446 frameshift mutation was identified in case 1, c.652G>A in case 2, p.Asp460 frameshift mutation in case 3, and p.Leu505Arg missense mutation in case 4. Although no hotspot was identified, several mutations were found to be localized near the transmembrane lesions of *PTCH1* in all four cases.

It is pertinent to note the diverse phenotypes shown in Table 1 and Fig 2. We think that these phenotypic divergences might indicate the existence of other gene mutations. First, we analyzed genes closely related to the Hh pathway, as these have wide range of biological functions and are likely to have impact on pathophysiology of Gorlin syndrome. We performed WES to simultaneously screen all exons of the 84 genes listed in Table 2. These 84 genes consisted of Hh ligands, receptors, transcription factors, and WNT, Hippo, and TGF/BMP signaling, all of which have a crosstalk with Hh signaling [36–39]. As expected, we observed several sequence variants. However, the consequences of these variants or mutations are usually difficult to assess in terms of their pathogenicity and require more detailed analyses. Thus we used open access programs to assess disease-causing potential of the DNA sequence variants.

First, we set a relatively high standard for variant candidates. The NGS coverage level often determines whether variant discovery can be made with a certain degree of confidence at particular base positions. It is generally recommended that for detecting human genome mutations, coverage should be at least from 10× to 30×. Therefore we preset the minimum sequence coverage to be 30.

In addition to coverage threshold, we applied MutationTaster2 and PolyPhen-2 analyses, which predict functional annotation of gene mutations, extract protein sequence annotations and structural attributes, and build conservation profiles. Through the use of two of these software programs, we were able to detect gene mutations which probably give rise to abnormal or malfunctioning proteins in three additional genes, *PTCH2*, *BOC*, *AFT4*, and *WNT9b*.

MutationTaster2 is a web-based application for rapid evaluation of the disease-causing potential of DNA sequence alterations. MutationTaster2 integrates information from different biomedical databases and employs established analytic tools[24,27,28].

We observed that some of the mutations in *PTCH2* were already reported to be disease causing, such as p.Arg175Leu and p.Arg74His in case 1[1,2]. We also detected p.Arg673Leu in *PTCH2* in case 3. These three mutations were determined as “disease causing” by MutationTaster2. Polyphen-2 showed p.Arg175Leu as “probably damaging” and p.Arg74His in case 1 and p.Arg673Leu in case 3 were “possibly damaging.” Thus, these are considered as possible disease-causing mutations.

*PTCH2* was reported to be a rare causative gene for Gorlin syndrome. Individuals who have mutations only in *PTCH2* showed relatively milder symptoms. It is intriguing that two out of four Gorlin patients had mutations in both *PTCH1* and *PTCH2*. These two genes have a redundant role because *PTCH2* gene has similar functions to those of *PTCH1*; however, *Ptch2*^*-/-*^ mice are viable and fertile, whereas *Ptch1*^-/-^ mice die at E9.5 with ectopic Hh signaling throughout the embryo [40,41]. Alfaro et al. showed that *Ptch1*^*–/–*^*; Ptch2*^*–/–*^neuralized embryoid bodies (NEBs) have a higher level of Shh pathway activation than *Ptch1*^*–/–*^NEBs [42]. It is conceivable that double mutations in *PTCH1* and *PTCH2* may lead to more severe symptoms.

p.Gly309Arg and p.Pro913Ser were detected in biregional Codon-binding protein (BOC), which positively regulates Shh signaling and promotes Shh-dependent cell fate specification and axon guidance [18,43]. The p.Gly309Arg mutation was predicted to be “probably damaging (0.998)” by polyphen-2. Cdo and Boc bind Shh through a high-affinity interaction with a specific fibronectin repeat that is essential for Hh activity [18]. *Cdo*^*−/−*^*; Boc*^−/−^ double mutants were reported to resemble *Gli2*^−/−^ mutants and lack the expression of Shh in the presumptive floor plate [44]. Although the biological significance of BOC is not well-characterized, to the best of our knowledge, BOC at least has a function as a Hh receptor.

PTCH2 and BOC are receptors of Hh and they regulate Hh pathway; therefore, they must have some effects on Hh signaling. It is noteworthy that all four patients had disease causing mutations in two of these Hh receptors.

We found a p.Ile1900Leu mutation in *FAT4* gene in case 1. We also found a p.Arg258His mutation in the *WNT9b* gene in case 2 and a p.Phe29Leu in the intron of *RUNX2* in case 3. *Fat4* is well known as a regulator of cell proliferation and planar cell polarity, and *Fat4*-null mice exhibit a short body with complicated morphological abnormalities, including abnormal orientation of inner ear hair cells, cystic dilation of kidney tubules, and defects in organ shapes [45]. *WNT9b* is the target gene of Hh and controls cell proliferation and cell polarity. *WNT9b* null mice display CLP and skeletal abnormalities in the upper jaw, which confirms that *WNT9b* is required for the proper development of the lip and palate [46]. An accumulating body of evidence suggests a crosstalk between Wnt and Hh [47]. Thus, mutations in *FAT4* and *WNT9b* may also play a supporting role in the development of Gorlin syndrome. The p.Phe29Leu in the intron of *RUNX2* gene was predicted to be “disease causing” by MutationTaster2. Several reports have indicated that mutations in introns could have some impact such as splicing aberrance or promotor enhancer dysfunction.

In this study, we focused on the genes that have been previously reported to have relationship with Hh signaling. Mutations were also detected in Hh unrelated genes, although its functional importance is unclear. We think that it is certainly possible that mutation in other genes may affect Hh signaling. Further investigation is required to elucidate the passivity. Only four individuals showed these mutations; however, if the number of samples increased, more significant information will be shown, and additional candidate gene related to Gorlin syndrome may be discovered.

Collectively, our findings indicate that NGS is essential to understand the genetic background of Gorlin syndrome. It may be important to identify mutations in Hh receptors other than *PTCH1*, such as *PTCH2* and *BOC*. Hereditary disorders are typically considered to be caused by a mutation on a single gene. However, to elucidate the genetic basis of a syndrome, WGS or WES is indispensable.
